# An exploratory analysis of missing data from the Royal Bank of Canada (RBC) Learn to Play – Canadian Assessment of Physical Literacy (CAPL) project

**DOI:** 10.1186/s12889-018-5901-z

**Published:** 2018-10-02

**Authors:** Christine Delisle Nyström, Joel D. Barnes, Mark S. Tremblay

**Affiliations:** Healthy Active Living and Obesity (HALO) Research Group, Children’s Hospital of Eastern Ontario Research Institute, 401 Smyth Road, Ottawa, ON K1H 8L1 Canada

**Keywords:** Children, Missing data, Physical literacy

## Abstract

**Background:**

Physical literacy comprises a range of tests over four domains (Physical Competence, Daily Behaviour, Motivation and Confidence, and Knowledge and Understanding). The patterns of missing data in large field test batteries such as those for physical literacy are largely unknown. Therefore, the aim of this paper was to explore the patterns and possible reasons for missing data in the Royal Bank of Canada Learn to Play–Canadian Assessment of Physical Literacy (RBC Learn to Play–CAPL) project.

**Methods:**

A total of 10,034 Canadian children aged 8 to 12 years participated in the RBC Learn to Play–CAPL project. A 32-variable subset from the larger CAPL dataset was used for these analyses. Several R packages (“Hmisc”, “mice”, “VIM”) were used to generate matrices and plots of missing data, and to perform multiple imputations.

**Results:**

Overall, the proportion of missing data for individual measures and domains ranged from 0.0 to 33.8%, with the average proportion of missing data being 4.0%. The largest proportion of missing data in CAPL was the pedometer step counts, followed by the components of the Physical Competence domain and the Children’s Self-Perception of Adequacy in and Predilection for Physical Activity subscales. When domain scores were regressed on five imputed subsets with the original subset as the reference, there were small and statistically detectable differences in the Daily Behaviour score (β = − 1.6 to − 1.7, *p* < 0.001). However, for the other domain scores the differences were negligible and statistically undetectable (β = − 0.01 to − 0.06, *p* > 0.05).

**Conclusions:**

This study has implications for other researchers or educators who are creating or using large field-based assessment measures in the areas of physical literacy, physical activity, or physical fitness, as this study demonstrates where problems in data collection can arise and how missing data can be avoided. When large proportions of missing data are present, imputation techniques, correction factors, or other treatment options may be required.

**Electronic supplementary material:**

The online version of this article (10.1186/s12889-018-5901-z) contains supplementary material, which is available to authorized users.

## Background

Physical literacy is defined as the “motivation, confidence, physical competence, knowledge and understanding to value and take responsibility for engagement in physical activities for life” [1]. The Royal Bank of Canada Learn to Play – Canadian Assessment of Physical Literacy (RBC Learn to Play–CAPL) was developed due to the shortage of objective physical literacy data, and aims to provide a reliable, feasible, and valid tool to assess physical literacy in children [2–4]. Within the CAPL there are four domains of measures (Physical Competence, Daily Behaviour, Motivation and Confidence, and Knowledge and Understanding), with participants receiving individual domain scores as well as an overall physical literacy score [5].

A Delphi process with international experts was used to develop the CAPL scoring system [6]. An overall physical literacy score was generated from multiple measures within the four domains. The Physical Competence and Daily Behaviour domains are more heavily weighted than the Knowledge and Understanding and Motivation and Confidence domains, due to the ability to objectively measure the former as well as due to some known limitations and biases within some of the protocols in the latter [2, 6]. Furthermore, in this process it was agreed that if a child was missing a single component within a domain or one entire domain, a domain score or overall physical literacy score could still be calculated using an algorithm designed to accommodate missing data and increase participant inclusion [3, 6]. The principle guiding these decisions was inclusivity, in order to ensure that as many children as possible were able to be included. This is important as the CAPL has numerous uses, including providing national surveillance, informing individual programs, providing evidence for resource allocation, and influencing policy decisions [4]. Thus, the inclusion of as many children as possible is extremely important. However, as with all decisions in research, there is the possibility that these decisions will influence the final results, as missing one particular component or domain could influence the results more than a child missing a different component or domain.

The problem of missing data is common to all types of studies, and it is important to understand the reasons why data are missing, to make sure the omission does not bias the results [7]. Missing data can be missing completely at random (i.e., there is no pattern in the missing data), missing at random (i.e., the missing data are significantly associated with the observed variables in the dataset), and missing not at random (i.e., the missing data are associated with the missing data pattern) [8]. As physical literacy is a relatively new area of measurement and CAPL is a new and unique dataset, it is important to understand the patterns and reasons for missing data. Because CAPL is a large test battery that encompasses various different measures, the results may be generalizable to other test batteries with multiple measures.

Thus, the aim of this paper was to explore the patterns and possible reasons for missing data in the RBC Learn to Play–CAPL project.

## Methods

### Study design and participants

The RBC Learn to Play–CAPL project was a Canada-wide cross-sectional study designed to measure the physical literacy of 8- to 12-year-old children [9]. Data were collected between 2014 and 2017 in 11 Canadian sites (Victoria, British Columbia; Lethbridge, Alberta; Calgary, Alberta; Winnipeg, Manitoba; North Bay, Ontario; Windsor, Ontario; Ottawa, Ontario; Trois-Rivières, Quebec; Antigonish, Nova Scotia; Halifax, Nova Scotia; and Charlottetown, Prince Edward Island). A convenience sampling method was utilized to recruit participants from various settings such as elementary schools, community centres, and after-school programs in order to have children of varying socioeconomic classes and levels of urbanization. A total of 10,034 children were included in the RBC Learn to Play–CAPL project; however, in this paper the number of children included varies by specific analyses due to the missing data.

Written informed consent was provided by parents or legal guardians, and verbal assent was provided by the children. The RBC Learn to Play–CAPL project was approved first by the Children’s Hospital of Eastern Ontario Research Ethics Board (the coordinating centre), and thereafter by each participating site’s institutional Research Ethics Board and participating school boards as required.

### CAPL protocol

The participant’s physical literacy was assessed using the published CAPL protocol [3, 6]. The CAPL manual [5], along with associated training materials, is freely available online in both English and French (https://www.capl-ecsfp.ca). Briefly, the CAPL comprises measures of four domains: Physical Competence, Daily Behaviour, Motivation and Confidence, and Knowledge and Understanding. The CAPL provides individual domain scores as well as an overall physical literacy score [3]. The maximum CAPL score is out of 100 points, with Physical Competence, Daily Behaviour, Knowledge and Understanding, and Motivation and Confidence having maximum scores of 32 points, 32 points, 18 points, and 18 points, respectively [3, 6]. A short description of each of the domains is provided below, with more details available from Tremblay et al. [9].

#### Physical Competence

The Physical Competence domain is comprised of objective measurements of physical fitness, motor performance, and anthropometrics. Physical fitness measures included cardiorespiratory fitness assessed using the Progressive Aerobic Cardiovascular Endurance Run (PACER) [10]; muscular strength measured using handgrip strength [11]; muscular endurance assessed using the abdominal plank test [12]; and flexibility measured using the sit-and-reach test [11]. The Canadian Agility and Movement Skill Assessment (CAMSA) [13] was used to evaluate motor fitness, and the anthropometrics assessed were body mass index (BMI) z-scores [14] and waist circumference [11].

#### Daily Behaviour

This domain incorporates objective and subjective assessments of physical activity and sedentary behaviour. Pedometers (YamaxDigiWalker SW-200, Yamax Corporation, Tokyo, Japan) were used to measure physical activity for seven consecutive days. Additionally, the children answered questions regarding the number of days they had engaged in at least 60 min of moderate- to vigorous-intensity physical activity (MVPA) in the past 7 days, and their daily screen time habits on weekdays and weekend days [5].

#### Motivation and Confidence

The Motivation and Confidence domain was evaluated with a questionnaire that included items taken from published instruments in order to assess the participant’s motivation and confidence to be physically active. Each participant’s adequacy in and predilection for physical activity was measured using the Children’s Self-Perception of Adequacy in and Predilection for Physical Activity (CSAPPA) subscales [15]. Additionally, the children answered questions regarding perceived benefits and barriers of physical activity [16] and questions about how their activity levels and skills compared to their peers [3].

#### Knowledge and Understanding

This domain utilized a questionnaire in order to evaluate the children’s knowledge and understanding of health-related terminology, the utilization of safety equipment in daily life, how to improve motor and fitness skills, and physical activity and sedentary behaviour guideline recommendations [17].

### Statistical analysis

A 32-variable subset of the larger CAPL dataset was used for these analyses, which consisted of age, gender, and the CAPL scores (25 components, four domain scores, and the overall physical literacy score) computed from the raw data in the CAPL dataset. The scoring algorithm with the missing protocol allowance had been previously applied to this data (see Additional file 1 for an example of the missing data scoring algorithm). Briefly, the CAPL algorithm for missing data involved the calculation of a re-weighted domain score when one protocol was missing, and/or the calculation of a re-weighted overall physical literacy score when one domain was missing. For example, the Physical Competence domain represented the sum of seven protocol scores (maximum value 160) divided by five (maximum value 32). If the sit-and-reach score (maximum value 8) was missing, the sum of the other six protocols (maximum value 152) was multiplied by 160 and divided by 152 (maximum value of 160). This re-weighed value was then divided by five (maximum value 32) so that it was mathematically comparable to the other Physical Competence scores [5]. Cohen’s *d* [18] was used to compare the scores in the RBC Learn to Play–CAPL project by the pedometer step counts score (missing vs. not missing).

**Fig. 1:**
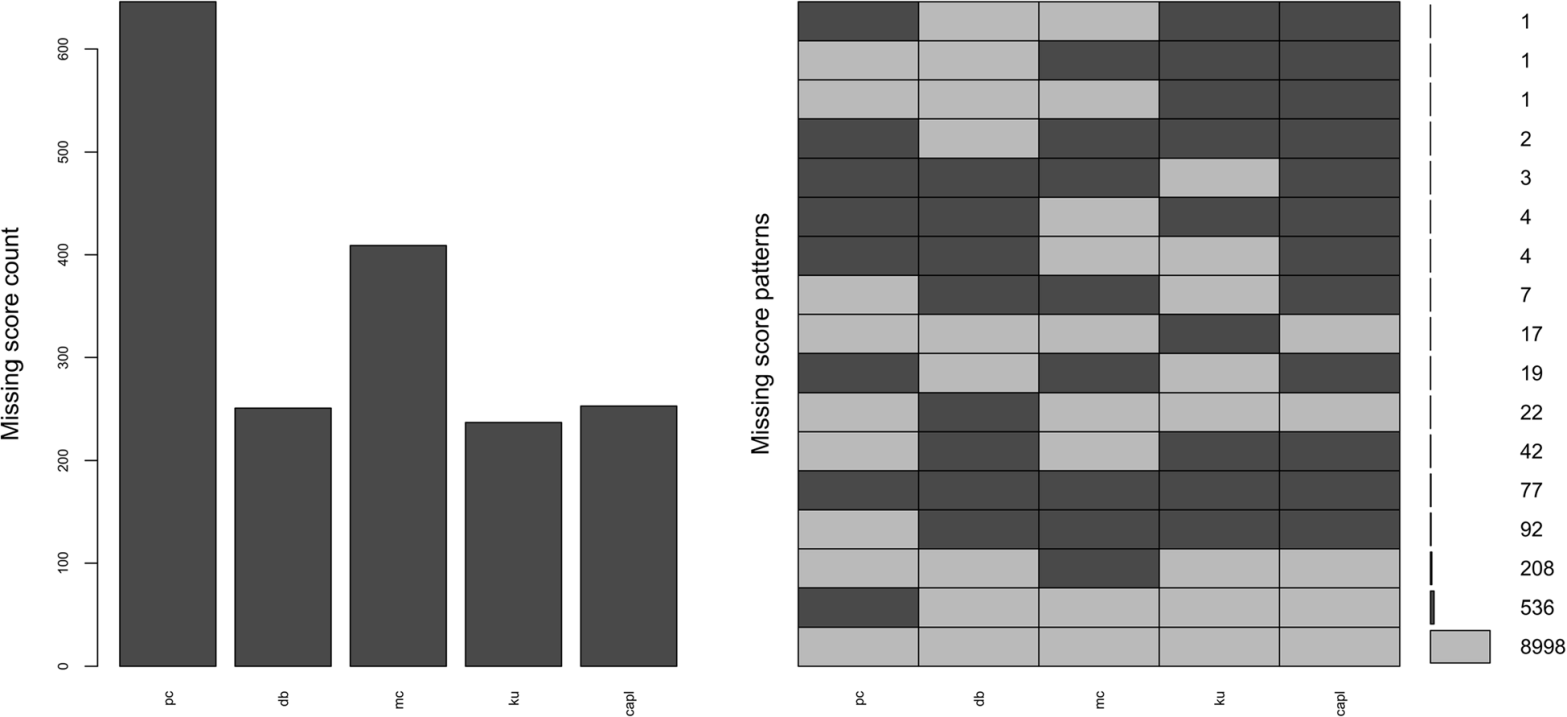
Frequency of all missing scores by domain and overall physical literacy (histogram on the left) and frequency of missing scores across scores by unique pattern (histogram on the right). *Note:* Dark grey bars and cells represent missing scores. In the histogram on the right, the numbers from the second row upward represent the number of missing scores per unique pattern. The bottom row (light grey cells across all columns) represents the number of complete scores across all domains and overall physical literacy. pc: Physical Competence score; db: Daily Behaviour score; mc: Motiviation and Confidence score; ku: Knowledge and Understanding score; capl: overall physical literacy score

**Fig. 2:**
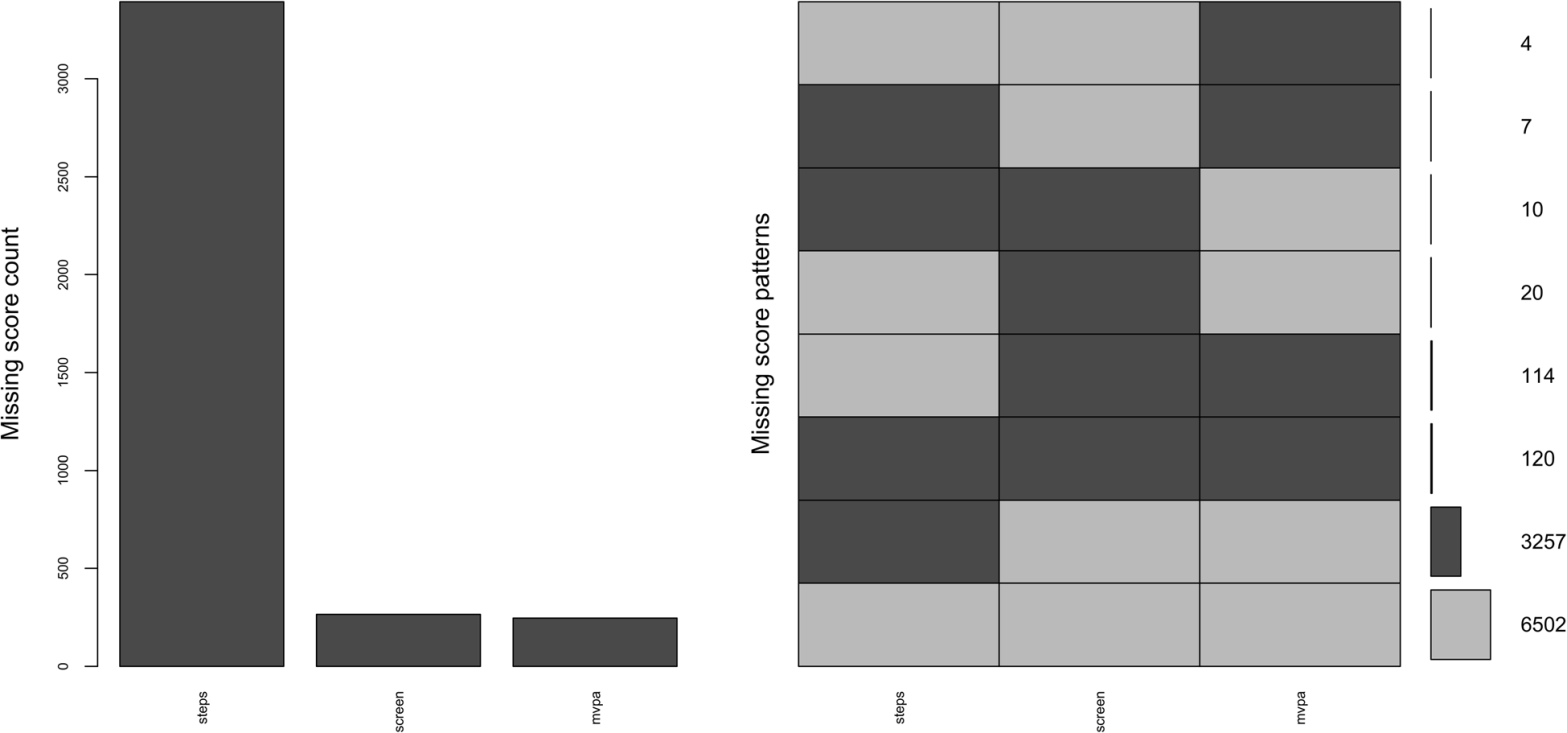
Frequency of all missing scores by Daily Behaviour protocol (histogram on the left) and frequency of missing scores across protocols by unique pattern (histogram on the right). *Note:* Dark grey bars and cells represent missing scores. In the histogram on the right, the numbers from the second row upward represent the number of missing scores per unique pattern. The bottom row (light grey cells across all columns) represents the number of complete scores across all Daily Behaviour protocols. Steps: pedometer score; screen: total screen time score; mvpa: self-reported moderate- to vigorous-intensity physical activity score

Several R packages (“Hmisc” [19], “mice” [20], “VIM” [21]) were used for all analyses. “Hmisc” [19] was used to generate matrices and plots of missing data (see Additional file 2), and “VIM” [21] was used to create plots for the frequencies and patterns of missing data (Figs. 1 and 2). The R package “rpart” [22] was used to run a recursive partitioning analysis using age, gender, height, weight, school grade, and site to fit a multivariable model on missing steps data (variable with the greatest missing data). The purpose of this analysis, which was run separately and prior to the multiple imputation analysis, was to determine whether the best predictor(s) of missing steps data was/were suggestive of data missing at random, which is an assumption of multiple imputation by chained equations. The R package “mice” [20] was used to run an exploratory analysis with multiple imputation by chained equations where five imputations were performed with 50 iterations per imputation. Predictive mean matching, proportional odds modelling, and polytomous logistic modelling were used for continuous variables, ordered categorical variables, and unordered categorical variables, respectively. Demographic variables (site, age, and gender) and raw protocol variables (e.g., sit-and-reach maximum value, pedometer steps for each day of the week, pedometer wear time for each day of the week) for each domain were imputed. CAPL scores were then computed from the values in each imputed dataset. These scores were combined with the original 32-variable dataset and identified by a grouping variable for comparison via regression analysis. R 3.4.4 (The R Foundation for Statistical Computing, Vienna, Austria) was used for all analyses.

## Results

Table 1 displays the proportion of missing data for the RBC Learn to Play - CAPL dataset. Overall, the proportion of missing data ranged from 0.0 to 33.8%, with the proportion of missing data being 4.0% on average. The largest source of missing data was step counts, at 33.8%. All of the components of the Physical Competence domain, as well as the overall Physical Competence domain score, had proportions of missing data, ranging from 3.6 to 6.4%. Furthermore, the CSAPPA predilection and adequacy subscales, and the overall domain score for Motivation and Confidence, had 4.0% missing data.

**Table 1:** Proportion of missing scores in the CAPL dataset stratified by domain after the application of CAPL missing data algorithm

Variable	Proportion of missing data (%)
Gender	0.0
Age	1.0
Physical Competence domain	
PACER 20 m shuttle run	6.4
CAMSA	5.4
Handgrip strength	3.6
Plank	4.2
BMI z-score	6.2
Waist circumference	6.4
Sit-and-reach flexibility	4.1
Physical Competence domain score	6.4
Daily Behaviour domain	
Step count (pedometer)	33.8
Self-reported sedentary time	2.6
Self-reported MVPA	2.4
Daily Behavior domain score	2.5
Knowledge and Understanding domain	
Physical activity comprehension and understanding	2.2
Minutes of daily PA guideline question	2.0
Screen time guideline question	2.0
Cardiorespiratory fitness definition	2.1
Muscular strength/endurance definition	2.0
Meaning of healthy question	1.8
Safety gear use during PA question	1.8
Improve sport skill question	2.4
Get in better shape question	2.3
Preferred leisure time activity question	2.0
Knowledge and Understanding domain score	2.4
Motivation and Confidence domain	
Activity level compared to peers question	1.7
Skill level compared to peers question	1.7
Benefits-to-barriers ratio	2.9
CSAPPA predilection scores	4.0
CSAPPA adequacy scores	4.0
Motivation and Confidence domain score	4.0
Overall CAPL score	2.5

*BMI* body mass index, *CAMSA* Canadian Agility and Movement Skill Assessment, *CAPL* Canadian Assessment of Physical Literacy, *CSAPPA* Children’s Self-Perception of Adequacy in and Predilection for Physical Activity, *MVPA* moderate- to vigorous-intensity physical activity, *PA* physical activity, *PACER* Progressive Aerobic Cardiovascular Endurance Run

Overall, there were 348 unique patterns of missing data within the subset of scores that we examined from the CAPL dataset. Figure 1 displays the frequencies and patterns of missing data for the domain scores and the overall physical literacy score within the RBC Learn to Play–CAPL project. A total of 8998 participants had enough data (i.e., had complete data or enough data to apply the missing data algorithm) to calculate the four domain scores and the overall physical literacy score. The Physical Competence domain had the highest proportion of missing scores (*n* = 646), with the Motivation and Confidence domain having the second highest proportion of missing domain scores (*n* = 409).

Figure 2 shows the frequencies and patterns of missing data for the Daily Behaviour domain components. A total of 6502 participants had complete data for all three components (pedometer, self-reported MVPA, and self-reported screen time). The most common pattern for missing data was for children missing step counts (*n* = 3257), with the next largest patterns for missing data being the children missing all three components for the Daily Behaviour domain (*n* = 120) or missing the two self-report questions (*n* = 114). The recursive partitioning analysis (missing step scores ~ age + gender + height + weight + school grade + site) suggested that site was the best predictor of missing data for step counts. Three of the 11 sites included in the RBC Learn to Play–CAPL project were missing step counts for 69.2% of their participants on average. Two four-site groupings had 38.2 and 21.4% of missing step counts for their participants on average, respectively.

Table 2 compares the scores in the project data by those missing versus not missing the pedometer scores (measure with the greatest missing data). Effect sizes for differences for all of the variables were considered negligible to small (range for Cohen’s *d*: 0.00 to 0.39).

**Table 2:** Comparison of scores in the RBC Learn to Play–CAPL project by either missing or not missing the pedometer step counts

Variable	Has steps			Missing steps			Cohen’s *d*
	n	Mean score	SD	n	Mean score	SD	
Age	6592	10.61	1.17	3337	10.58	1.23	0.03
Physical Competence domain							
PACER 20 m shuttle run (/42)	6362	19.79	9.23	3031	20.07	9.97	−0.03
Overall obstacle course score (CAMSA) (/42)	6428	31.13	5.70	3060	30.57	6.07	0.10
Handgrip strength (/17)	6525	8.77	4.24	3143	9.11	4.29	−0.08
Plank (/17)	6498	7.89	4.59	3111	7.65	4.67	0.05
BMI z-score (/17)	6390	14.07	4.33	3022	13.91	4.46	0.04
Waist circumference (/17)	6377	11.72	4.60	3018	11.54	4.66	0.04
Sit and reach (/8)	6503	5.03	2.39	3117	4.72	2.37	0.13
Physical Competence domain score (/32)	6385	19.68	4.31	3003	19.44	4.57	0.05
Daily Behaviour domain							
Step count (pedometer) (/21)	6640	10.28	4.98	–	–	–	–
Self-reported sedentary time (/8)	6506	5.15	2.91	3264	4.94	3.00	0.07
Self-reported MVPA (/3)	6522	2.18	0.90	3267	2.12	0.96	0.07
Daily Behaviour domain score (/32)	6526	17.58	6.30	3257	20.51	9.58	−0.39
Knowledge and Understanding domain							
Physical activity comprehension and understanding (/5)	6542	3.62	1.44	3272	3.55	1.48	0.05
Minutes of daily PA guideline question (/1)	6554	0.64	0.48	3279	0.60	0.49	0.09
Screen time guideline question (/1)	6554	0.14	0.35	3283	0.16	0.37	−0.05
Cardiorespiratory fitness definition (/1)	6549	0.58	0.49	3273	0.54	0.50	0.08
Muscular strength/endurance definition (/1)	6552	0.76	0.43	3277	0.71	0.45	0.11
Meaning of healthy question (/5)	6563	4.00	0.93	3293	3.89	0.97	0.12
Safety gear use during PA question (/1)	6563	0.34	0.28	3293	0.30	0.29	0.16
Improve sport skill question (/1)	6534	0.51	0.50	3264	0.47	0.50	0.08
Get in better shape question (/1)	6535	0.81	0.39	3269	0.78	0.42	0.08
Preferred leisure time activity question (/1)	6551	0.74	0.44	3284	0.70	0.46	0.08
Knowledge and Understanding domain score (/18)	6533	12.15	2.67	3264	11.70	2.85	0.16
Motivation and Confidence domain							
Activity level compared to peers question (/1)	6574	0.72	0.21	3291	0.71	0.22	0.05
Skill level compared to peers question (/1)	6574	0.67	0.23	3289	0.67	0.24	0.00
Benefits-to-barriers ratio (/4)	6508	1.60	1.14	3238	1.53	1.22	0.06
CSAPPA predilection scores (/6)	6460	4.84	0.96	3168	4.73	1.02	0.11
CSAPPA adequacy scores (/6)	6460	4.71	0.91	3168	4.65	0.94	0.06
Motivation and Confidence domain score (/18)	6458	12.55	2.68	3167	12.30	2.83	0.09
Overall CAPL score (/100)	6529	61.95	10.78	3252	64.13	14.57	−0.17

*BMI* body mass index, *CAMSA* Canadian Agility and Movement Skill Assessment, *CAPL* Canadian Assessment of Physical Literacy, *CSAPPA* Children’s Self-Perception of Adequacy in and Predilection for Physical Activity, *MVPA* moderate- to vigorous-intensity physical activity, *PA* physical activity, *PACER* Progressive Aerobic Cardiovascular Endurance Run, *RBC* Royal Bank of Canada, *SD* standard deviation

When the Daily Behaviour score was regressed on a grouping variable (nominal variable from 0 to 5, where 1 to 5 represented the five imputed datasets and zero represented the original dataset, which served as the reference), the scores were 1.6–1.7 units lower on average (*p* < 0.001) compared to the Daily Behaviour score in the original dataset. When expressed as Cohen’s *d* coefficients, this represents effect sizes ranging from 0.23 to 0.25. Using the same regression analysis on the other domain scores (Physical Competence, Motivation and Confidence, and Knowledge and Understanding) resulted in very small differences in domain scores between the imputed datasets and the original dataset. There were no statistically significant differences for Physical Competence scores in the imputed datasets (0.04 to 0.06 units lower), Motivation and Confidence scores in the imputed datasets (0.03 units lower), and Knowledge and Understanding scores in the imputed datasets (0.01 units lower).

## Discussion

Missing data is a common problem in research, irrespective of study design [7]. The largest proportion of missing data in the RBC Learn to Play–CAPL project was the objectively assessed physical activity (pedometer step counts), distantly followed by the components of the Physical Competence domain and the CSAPPA subscales.

It has been found that compliance for US children wearing pedometers ranges from 46 to 98% [23], which is in line with the results observed in the RBC Learn to Play–CAPL project, where the compliance rate for the pedometers was 66.2%. However, even though our compliance rates are in line with previous research, there is still a large proportion of missing data that needs to be addressed and treated appropriately.

In this exploratory analysis of missing data within the RBC Learn to Play–CAPL project, our recursive partitioning analysis suggests that the largest influence on missing pedometer data was the test site and not demographic characteristics such as age, gender, weight, or height. Thus, it is likely that our missing pedometer data was missing at random, which indicates that the missing data are related to observed variables and not unobserved variables [7]. Therefore, the suggested method to handle the missing data would be multiple imputation [7]. Advantages of multiple imputation include the ability to provide unbiased estimates using only a few imputed datasets, and the capacity to compare the imputed and observed values [24]. However, the end user of the data needs to be considered before a decision on how to handle the missing data is made. If CAPL or other large test batteries with multiple measures are being used for research purposes, it is advised that the data be analyzed to see if similar missing data patterns are observed. If they are, and the data are missing at random, multiple imputation may be necessary. However, large assessment batteries such as CAPL are often used by physical education teachers to inform students and parents on the child’s physical literacy development. Therefore, using techniques such as multiple imputation would not be feasible, as those outside of research would not know or be able to easily apply this technique.

Our exploratory missing data analysis showed that the imputed datasets Daily Behaviour scores were 1.6 to 1.7 units lower on average when compared to the domain score in the original CAPL data frame, which corresponds to a small difference when expressed as a Cohen’s *d* (0.23–0.25). This is evidence that the existing scoring algorithm is not greatly influenced by the missing scores and therefore, the small difference in scoring seems to be an acceptable trade-off in exchange for a more inclusive assessment battery, with simple missing data procedures. However, in future versions of the CAPL it would be worth considering to not calculate the Daily Behaviour domain score for those missing pedometer step counts, as negligible differences were observed between overall CAPL scores for those with and without the Daily Behaviour domain score (data not shown).

Other sources of missing data within the RBC Learn to Play–CAPL project were the components within the Physical Competence domain and the CSAPPA subscales. The higher degree of missing data within the Physical Competence domain (range from 3.6 to 6.4%) might be due to the perceived difficulty and invasiveness of the tests. This domain encompassed five physical fitness tests (handgrip strength, sit and reach, plank, CAMSA, and PACER), and the degree of missing data increased with the intensity of the tests (e.g., 3.6% for handgrip and 6.4% for the PACER). Furthermore, for the anthropometrics, the proportion of missing data was 6.2% for BMI and 6.4% for waist circumference. However, our exploratory missing data analysis showed that the imputed datasets had Physical Competence scores that differed negligibly on average and were statistically undetectable when compared to the domain score in the original CAPL data frame.

The final large source of missing data in the RBC Learn to Play–CAPL project was the CSAPPA subscales. The amount of missing data for this component (4.0%) could possibly be due to the design of the questionnaire. The CSAPPA subscales are based upon the Harter format [25], where each question is comprised of two matched contrasting statements (e.g., some kids can’t wait to play active games after school BUT other kids would rather do something else after school). The participant is then asked to select which statement fits them best and if it is ‘really true for me’ or ‘sort of true for me’. As this type of question format has been found to be difficult for both children and adults [26, 27], it is possible that the children participating in the RBC Learn to Play–CAPL project did not fully understand how to answer these questions, and therefore skipped them. However, our exploratory missing data analysis showed that the imputed datasets had Motivation and Confidence scores that differed negligibly on average and were statistically undetectable when compared to the domain score in the original CAPL data frame.

Changes have been made to the CAPL testing process that it is hoped will lead to fewer instances of missing data. The CAPL is an extensive battery comprised of 25 components split into four domains. Due to the extensiveness of the CAPL battery, factor analyses were conducted in order to create a shorter and more theoretically aligned version of the CAPL [28]. Using confirmatory factor analysis, Gunnell et al. [28] found that the CAPL could be reduced to 14 components, and this new version was entitled CAPL-2. CAPL-2 consists of the same four domains as CAPL; however, the number of tests in each domain is greatly reduced [29]. For instance, the Physical Competence domain is now comprised only of the PACER, CAMSA, and plank. Even though these three components had a relatively high degree of missing data in CAPL (between 4.2 and 6.4%), it is thought that due to the reduction in tests, it will be less daunting for the children to complete and therefore there will be less missing data. Furthermore, the CSAPPA subscales are still included within the CAPL-2 battery; however, with the fewer number of subscale items (6 in CAPL-2 vs. 17 in CAPL), it is believed that evaluators will have more time to explain to the children how to properly fill in the questions as well as answer any questions the participants may have. Thus, it is hoped that the missing data for this component will be reduced in CAPL-2; future research should explore missing data in CAPL-2. Finally, the pedometer step counts, which were the largest source of missing data in CAPL (at 33.8%), are still included in CAPL-2. As site may be one of the strongest predictors for missing data for the pedometer, we recommend stressing to evaluators the importance of collecting complete data in participants. Another solution is to suspend the missing protocol rule for this domain so that if pedometer step counts are missing, no Daily Behaviour score is calculated. Even though missing data are inevitable, especially with objectively measured physical activity, a level of 34% is much too high.

To the best of our knowledge, this is the only study that has investigated the patterns of missing data in a large test battery for physical literacy consisting of a range of physical activity- and physical fitness-related components. This study has implications for other researchers or educators who are creating or using large field-based assessment measures in the area of physical literacy, physical activity, or physical fitness, as it demonstrates where problems in data collection may arise. Accordingly, measures can be put in place to avoid missing data before data collection begins. Examples of measures that can be put in place to help avoid missing data include: dividing fitness testing into two or more sessions depending on the number of tests; providing children with an alternative time for measuring body composition without their peers around; educating evaluators on the importance of having complete data; and, if utilizing questions that are using the Harter format [25], taking time to carefully explain to the participants how to correctly answer them.

## Conclusions

We found that the largest sources of missing data in the RBC Learn to Play–CAPL project were pedometer step counts, followed by the components of the Physical Competence domain, and the CSAPPA subscales. Furthermore, we observed that the scoring algorithm used to calculate CAPL domain scores and the overall physical literacy score were not greatly influenced by the largest source of missing data (pedometer step counts), providing support for the use of the missing data algorithm.

## Additional files

Additional file 1: The Canadian Assessment of Physical Literacy (CAPL) comprehensive scoring system and an example of CAPL’s scoring algorithm with the missing protocol allowance. (DOCX 506 kb)

Additional file 2: Examples of the the plots generated using the “Hmisc” R package for the CAPL dataset. (DOCX 417 kb)

## Abbreviations

BMI: Body mass index
CAMSA: Canadian Agility and Movement Skill Assessment
CAPL: Canadian Assessment of Physical Literacy
CSAPPA: Children’s self-perception of adequacy in and predilection for physical activity
MVPA: Moderate- to vigorous-intensity physical activity
PACER: Progressive aerobic cardiovascular endurance run
RBC: Royal Bank of Canada
