# Supplementary material for: An exploratory analysis of missing data from the Royal Bank of Canada (RBC) Learn to Play – Canadian Assessment of Physical Literacy (CAPL) project
Source: BMC Public Health. 2018 Oct 2;18(Suppl 2):1046. doi: 10.1186/s12889-018-5901-z (PMC6167773; doi:10.1186/s12889-018-5901-z)
Supplement: Supplementary file 1 — The Canadian Assessment of Physical Literacy (CAPL) comprehensive scoring system and an example of CAPL’s scoring algorithm with the missing protocol allowance. (DOCX 506 kb) [file 12889_2018_5901_MOESM1_ESM.docx]

**Figure S1.** Canadian Assessment of Physical Literacy comprehensive scoring system.


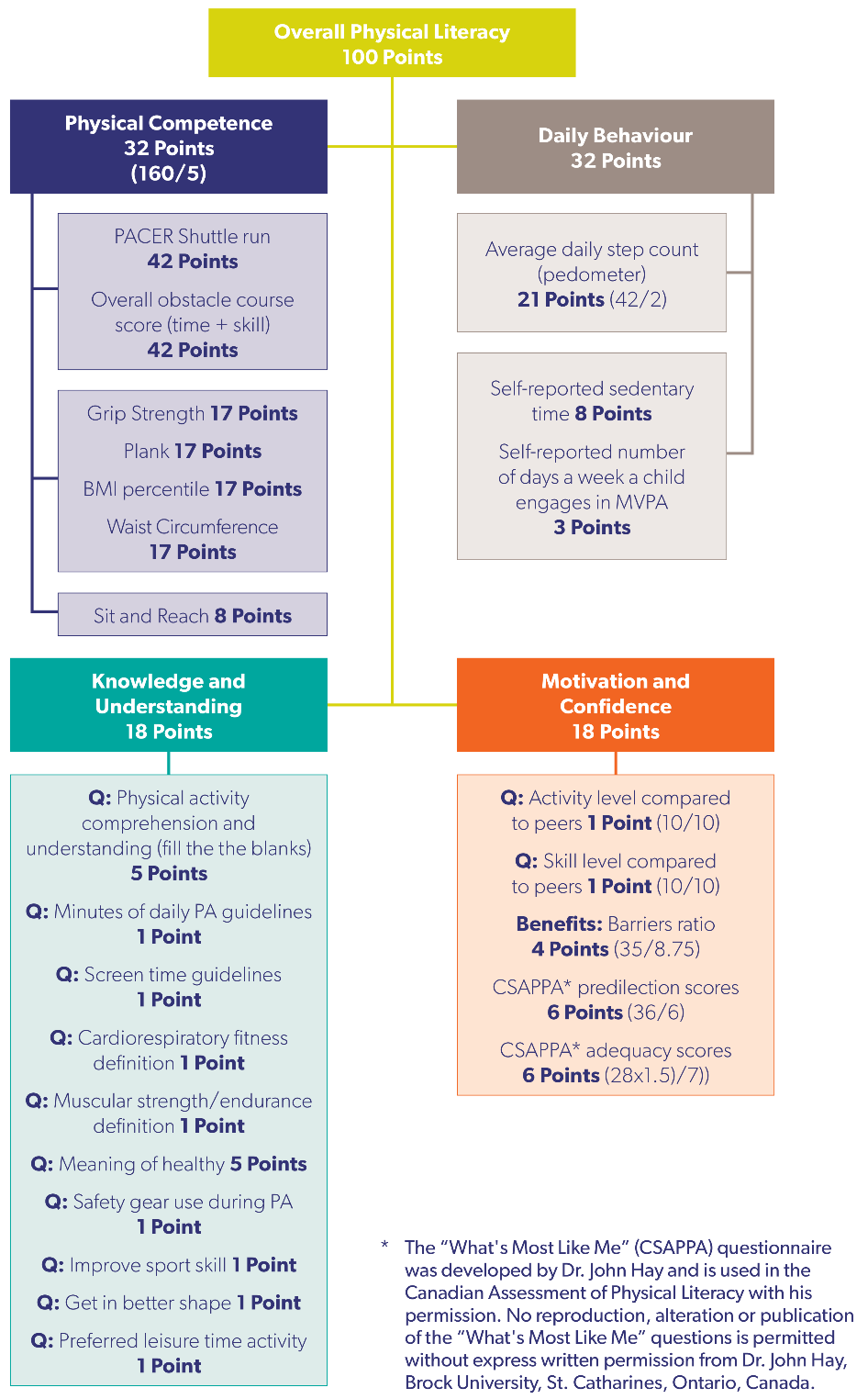


BMI: body mass index; CSAPPA: Children’s Self-Perception of Adequacy in and Predilection for Physical Activity; MVPA: moderate- to vigorous-intensity physical activity; PA: physical activity; PACER: Progressive Aerobic Cardiovascular Endurance

**Table S1.** Example of the CAPL scoring algorithm with the missing protocol allowance for one participant.

| **Score** | **Complete scores** | **Missing handgrip score** | **Missing Physical Competence domain** |
| --- | --- | --- | --- |
| Age | 10.1 | 10.1 | 10.1 |
| Gender | Girl | Girl | Girl |
| Sit-and-reach flexibility | 2 | 2 |  |
| Handgrip strength | 3 |  |  |
| PACER | 7 | 7 |  |
| Plank | 3 | 3 |  |
| BMI z-score | 5 | 5 |  |
| Waist circumference | 3 | 3 |  |
| CAMSA | 13.5 | 13.5 |  |
| Physical Competence domain score | 7.3 | 7.5 |  |
| Step count (pedometer) | 8 | 8 | 8 |
| Self-reported sedentary time | 0 | 0 | 0 |
| Self-reported MVPA | 1 | 1 | 1 |
| Daily Behaviour domain score | 9 | 9 | 9 |
| Activity level compared to peers question | 0.6 | 0.6 | 0.6 |
| Skill level compared to peers question | 0.4 | 0.4 | 0.4 |
| Benefits-to-barriers ratio | 0.5 | 0.5 | 0.5 |
| CSAPPA predilection score | 3.2 | 3.2 | 3.2 |
| CSAPPA adequacy score | 3 | 3 | 3 |
| Motivation and Confidence domain score | 7.7 | 7.7 | 7.7 |
| Physical activity comprehension and understanding | 1 | 1 | 1 |
| Minutes of daily PA guideline question | 1 | 1 | 1 |
| Screen time guideline question | 0 | 0 | 0 |
| Cardiorespiratory fitness definition | 1 | 1 | 1 |
| Muscular strength/endurance definition | 1 | 1 | 1 |
| Meaning of healthy question | 3 | 3 | 3 |
| Safety gear use during PA question | 0.27 | 0.27 | 0.27 |
| Improve sport skill question | 0 | 0 | 0 |
| Get in better shape question | 1 | 1 | 1 |
| Preferred leisure time activity question | 0 | 0 | 0 |
| Knowledge and Understanding domain score | 8.3 | 8.3 | 8.3 |
| Overall CAPL score | 32.3 | 32.5 | 36.8 |

BMI: body mass index; CAMSA: Canadian Agility and Movement Skill Assessment; CAPL: Canadian Assessment of Physical Literacy; CSAPPA: Children’s Self-Perception of Adequacy in and Predilection for Physical Activity; MVPA: moderate- to vigorous-intensity physical activity; PA: physical activity; PACER: Progressive Aerobic Cardiovascular Endurance

Note: Red cells represent missing scores and yellow cells represent changes to scores.
