# Supplementary material for: An exploratory analysis of missing data from the Royal Bank of Canada (RBC) Learn to Play – Canadian Assessment of Physical Literacy (CAPL) project
Source: BMC Public Health. 2018 Oct 2;18(Suppl 2):1046. doi: 10.1186/s12889-018-5901-z (PMC6167773; doi:10.1186/s12889-018-5901-z)
Supplement: Supplementary file 2 — Examples of the the plots generated using the “Hmisc” R package for the CAPL dataset. (DOCX 417 kb) [file 12889_2018_5901_MOESM2_ESM.docx]

**Figure S2a.** Plot of missing data proportions in ascending order.


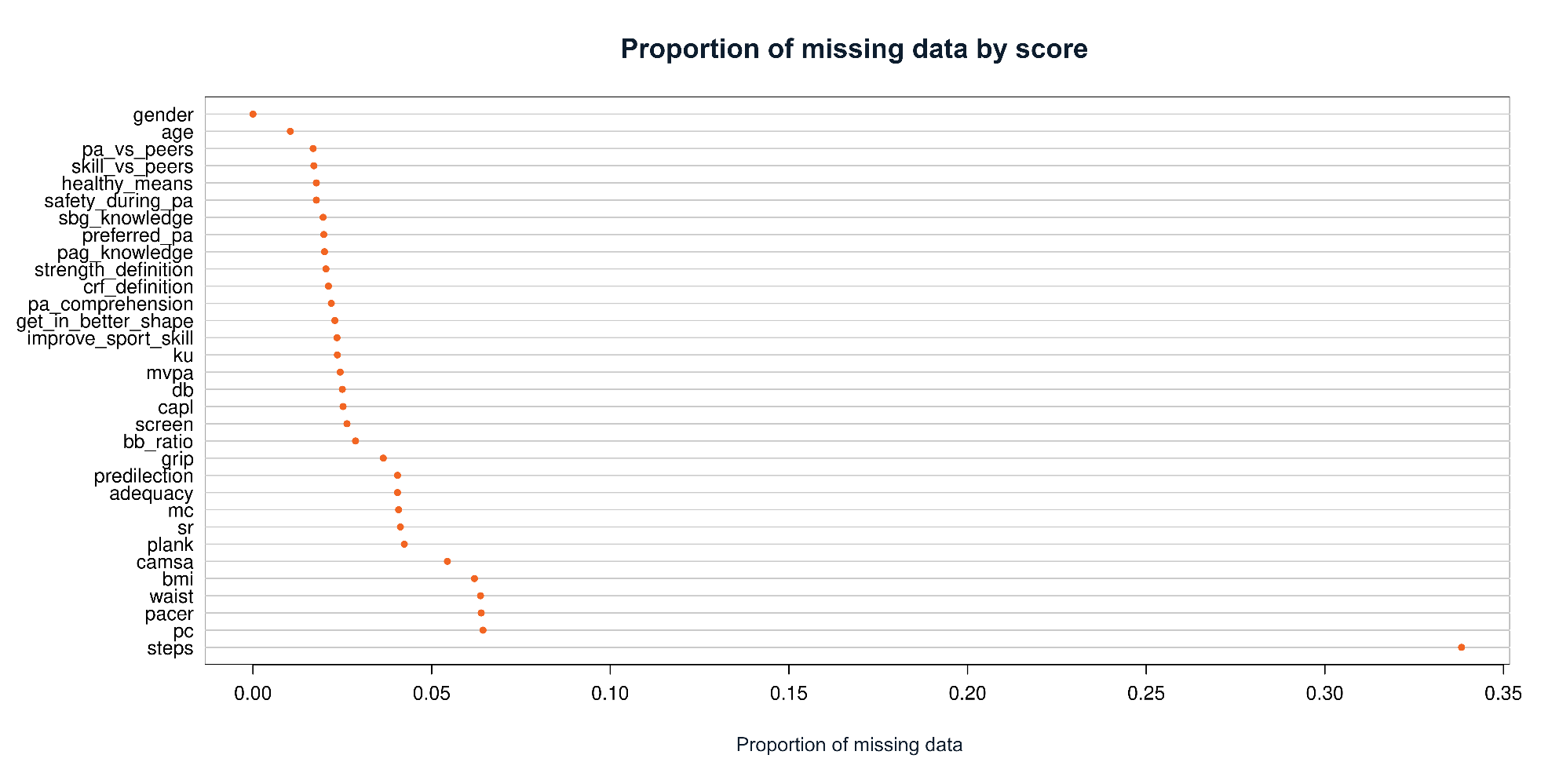


bb: benefits-to-barriers ratio; bmi: body mass index; camsa: Canadian Agility and Movement Skill Assessment; capl: Canadian Assessment of Physical Literacy score; crf: cardiorespiratory fitness; db: Daily Behaviour domain; grip: handgrip strength; ku: Knowledge and Understanding domain; mc: Motivation and Confidence domain; mvpa: moderate- to vigorous-intensity physical activity; pa: physical activity; pacer: Progressive Aerobic Cardiovascular Endurance; pag: physical activity guidelines; pc: Physical Competence domain; sbg: sedentary behaviour guidelines; sr: sit and reach; waist: waist circumference

Note: This plot was generated using the “Hmisc” R package [19].

**Figure S2b**. Visualization of how missing data in the CAPL dataset cluster by variable groupings.


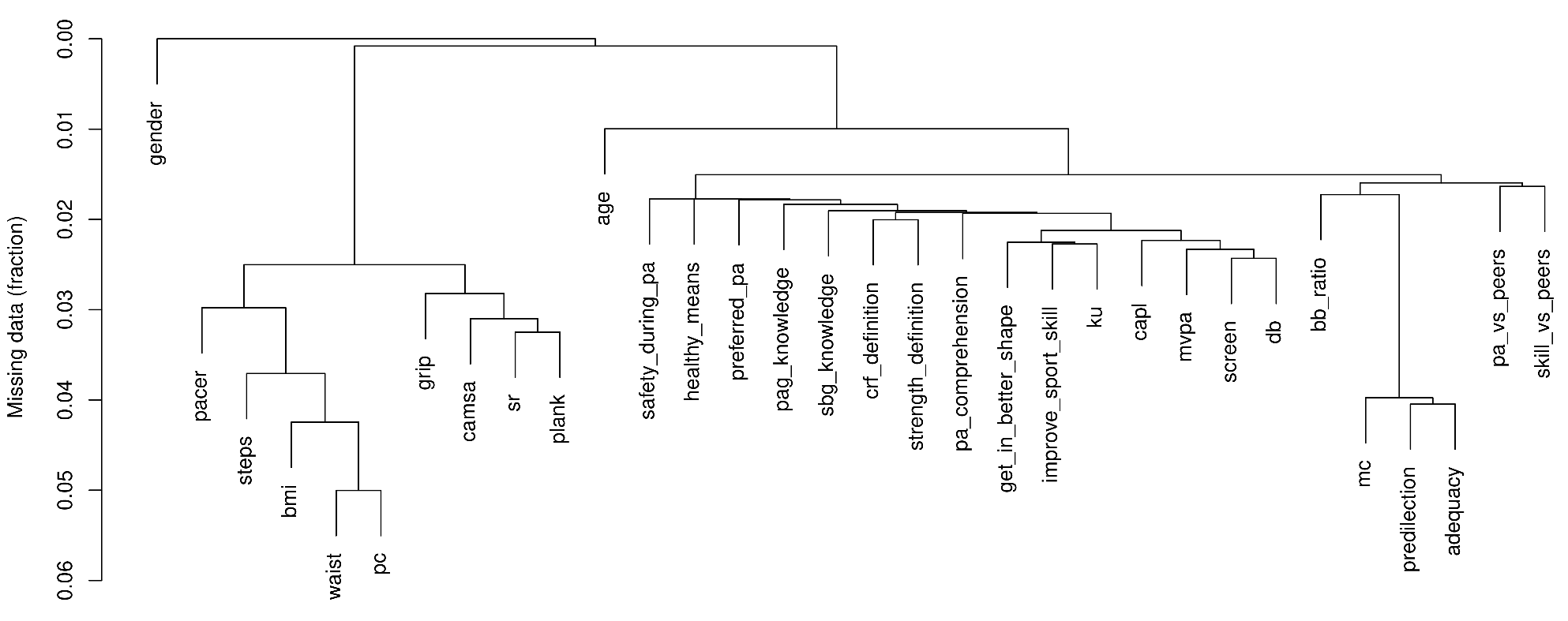


bb: benefits-to-barriers ratio; bmi: body mass index; camsa: Canadian Agility and Movement Skill Assessment; capl: Canadian Assessment of Physical Literacy score; crf: cardiorespiratory fitness; db: Daily Behaviour domain; grip: handgrip strength; ku: Knowledge and Understanding domain; mc: Motivation and Confidence domain; mvpa: moderate- to vigorous-intensity physical activity; pa: physical activity; pacer: Progressive Aerobic Cardiovascular Endurance; pag: physical activity guidelines; pc: Physical Competence domain; sbg: sedentary behaviour guidelines; sr: sit and reach; waist: waist circumference

Note: This plot was generated using the “Hmisc” R package [19].
